# Cross-National Associations Among Cyberbullying Victimization, Self-Esteem, and Internet Addiction: Direct and Indirect Effects of Alexithymia

**DOI:** 10.3389/fpsyg.2020.01368

**Published:** 2020-06-11

**Authors:** Sebastian Wachs, Alexander T. Vazsonyi, Michelle F. Wright, Gabriela Ksinan Jiskrova

**Affiliations:** ^1^Department of Educational Sciences, University of Potsdam, Potsdam, Germany; ^2^Department of Family Sciences, University of Kentucky, Lexington, KY, United States; ^3^Child Study Center, Department of Psychology, Pennsylvania State University, University Park, PA, United States; ^4^Faculty of Social Studies, Masaryk University, Brno, Czechia; ^5^School of Social Work, Virginia Commonwealth University, Richmond, VA, United States

**Keywords:** cyberbullying victimization, alexithymia, self-esteem, internet addiction, adolescents

## Abstract

The relationship among cyberbullying victimization, lower self-esteem, and internet addiction has been well-established. Yet, little research exists that explains the nature of these associations, and no previous work has considered the inability to identify or describe one’s emotions, namely, alexithymia, as a potential mediator of these links. The present study sought to investigate the indirect effects of cyberbullying victimization on self-esteem and internet addiction, mediated by alexithymia. The sample consisted of 1,442 participants between 12 and 17 years (*M*_age_ = 14.17, *SD* = 1.38, 51.5% male) from Germany, the Netherlands, and the United States. Results showed a direct relationship between cyberbullying victimization and self-esteem and an indirect association mediated by alexithymia in the Dutch sample. However, in the German and U.S. samples, only an indirect relationship via alexithymia, but not a direct effect of cyberbullying victimization on self-esteem, was found. Consistent across the three country samples, cyberbullying victimization and internet addiction were directly and also indirectly associated via alexithymia. In sum, findings indicate that alexithymia might help better understand which detrimental effects cyberbullying victimization has on adolescent psychological health. Thus, cyberbullying prevention programs should consider implementing elements that educate adolescents on the ability to identify and describe their own emotions.

## Introduction

Cyberbullying is an umbrella term for any aggressive behavior (i.e., harassment, denigration, outing, and exclusion) repeatedly performed by individuals or groups through information and communication technologies (ICTs) intended to inflict harm or discomfort on a person or group (Smith et al., 2008; Brosowski et al., 2018). Over the last years, research has well documented the detrimental effects cyberbullying victimization can have on adolescents’ wellbeing and psychological health, such as lower self-esteem and higher level of addictive behaviors (e.g., Kowalski et al., 2014). These consequences constitute a serious risk for adolescents’ development because low self-esteem during adolescence predicts higher problem behavior and lower economic success later in life (Trzesniewski et al., 2006; Orth et al., 2008) and addictive behaviors emerging in adolescence often retain into adulthood (Englund et al., 2013). However, only a few studies have tried to identify which factors explain the mechanism that underlies the relationship of cyberbullying victimization, lower self-esteem, and higher level of addiction. Alexithymia might play an important role in understanding these associations. On one hand, alexithymia has been shown to be related to higher risk of traditional and cyber bullying victimization (e.g., Guzzo et al., 2014; Aricak and Ozbay, 2016), lower self-esteem (e.g., Sasai et al., 2010), and higher level of internet addiction (e.g., Dalbudak et al., 2013). On the other hand, alexithymia has been found to mediate the relationships between bullying victimization and negative psychological health (Guzzo et al., 2014), traumatic experiences and internet addiction (Yates et al., 2012), and bullying victimization and internalizing/externalizing symptoms (Prino et al., 2019). Yet, no research has been conducted to investigate the relationship among cyberbullying victimization, alexithymia, self-esteem, and internet addiction in one study. To this end, the present study investigates (a) whether cyberbullying victimization is indirectly associated with lower self-esteem via greater alexithymia and (b) whether cyberbullying victimization is indirectly related to internet addiction via greater alexithymia. The findings might help to identify cyberbullied adolescents’ needs and to understand which variables contribute to their psychological maladjustment. In addition, the results might also provide evidence for the development of precise intervention programs for cyberbullied victims on including elements that educate adolescents to articulate and read their own emotions. Finally, findings might inform parents/educators teachers, and school counselors on how to support cyberbullied adolescents.

## Investigating the Associations Among Cyberbullying Victimization, Self-Esteem, Internet Addiction, and Alexithymia From a Trauma Theoretical Perspective

Humans instinctually attempt to heal after traumatic experiences, but sometimes trauma can impact them in ways that are not understood, recognized, or manifested for many years (Herman, 1992; Levine, 2005). Trauma can increase hypervigilance, anxiety, distress, and disrupt people’s ability to connect with themselves, their families, and other individuals in their lives, and it can overwhelm their ability to handle everyday events (van der Kolk, 2003; Ford and Courtois, 2009). Furthermore, traumatic experiences can undermine self-esteem, self-confidence, and feelings of well-being (Levine, 2005). Survivors of trauma might find it difficult to establish boundaries with others, handle conflict, denigrate themselves and others, mishandle or not recognize cues of danger, and succumb to strong personalities and authority figures (Herman, 1992). Cyberbullying victimization functions as a type of traumatic experience in adolescents’ lives (Baldry et al., 2019; Sjursø et al., 2019). It has the potential to diminish their well-being, lower their self-esteem, disrupt their ability to handle emotions, and increase their vulnerability to problematic behaviors. In this section, we review from a trauma theoretical perspective key evidence for a relationship among cyberbullying victimization (traumatic experience), adolescents’ self-esteem (mental health), problem behaviors (e.g., Internet addiction), and regulation disorders in emotion (e.g., alexithymia).

### Understanding the Relationship Among Cyberbullying Victimization, Self-Esteem, and Alexithymia

Self-esteem is a person’s overall subjective emotional evaluation of self-worth (Rosenberg, 1965). Individuals with low self-esteem are characterized by little or no awareness of their feelings and needs and have a negative outlook on life (Rosenberg, 1965). There are a number of reasons why cyberbullying victimization might lead to lower self-esteem. Particularly during adolescence, peer acceptance and relationships are important to adolescents’ self-esteem development (Tetzner et al., 2017). However, adolescents who are cyberbullied, namely, excluded, harassed, and/or denigrated repeatedly, often withdraw offline from peers and family (Ortega Barón et al., 2019); they also experience higher levels of peer rejection as compared with adolescents who do not experience cyberbullying victimization (Wright and Wachs, 2019). Therefore, cyberbullied adolescents may socialize less with others and therefore experience fewer positive interactions which in turn contributes to an inability to develop a positive sense of self. At the same time, they become insecure and establish feelings of helplessness, inferiority, and resentment (Ortega et al., 2012). Thus, it is reasonable to suggest that being cyberbullied by peers has a negative impact on adolescent self-esteem. Indeed, some studies have shown that adolescents are adversely affected by cyberbullying victimization, particularly low self-esteem (Brighi et al., 2012; Olweus, 2012; Brewer and Kerslake, 2015; Wachs et al., 2016; Palermiti et al., 2017; Lei et al., 2019).

The term alexithymia has been coined by Sifneos (1973). Although every human being has feelings, a number of individuals show an inability to read and describe their subjective emotions, which is described as alexithymia. Until now, there is a vivid debate on whether alexithymia should be considered a personality state (i.e., Chinet et al., 1998) or trait (i.e., Luminet et al., 1999), which has resulted in distinguishing between at least two types of alexithymia. While one type is posited to be a stable personality trait (primary alexithymia), the second type is considered to be a state reaction evoked by stressful situations and traumatic experiences (secondary alexithymia; Eichhorn et al., 2014; Karukivi and Saarijärvi, 2014). There is good reason to suggest that secondary alexithymia might be related to cyberbullying victimization. Cyberbullying victimization can be understood as a traumatic experience for many adolescents (Baldry et al., 2019). In these cases, alexithymic symptoms might therefore occur as a reaction to the stress caused by cyberbullying and could be understood as a psychological mechanism by which adolescents try to suppress and deny painful emotions resulting from cyberbullying victimization. Indeed, there is some empirical research that has revealed a positive relationship between alexithymia and physical, verbal, relational, and cyber bullying victimization (Garisch and Wilson, 2010; Guzzo et al., 2014; Aricak and Ozbay, 2016; Prino et al., 2019).

Despite dysfunction in emotional awareness, core characteristics of alexithymia are a lack of social attachment, poor interpersonal relationships, and social skills (Sifneos, 1973; Eichhorn et al., 2014; Karukivi and Saarijärvi, 2014). As mentioned earlier, social attachment and interpersonal relationships play a crucial role in developing self-esteem among adolescents (Tetzner et al., 2017). If alexithymic individuals, however, struggle with interpersonal relating, this might have a negative effect on their self-esteem. There is some empirical evidence to support this proposal for young adults (Yelsma, 1995; De Berardis et al., 2009; Sasai et al., 2010). However, this relationship has not often been studied thoroughly among adolescents. Although initial research in a small sample of high school students has shown a negative relationship between alexithymia and self-esteem (Sayar et al., 2005), additional work in this area is needed including larger samples of adolescents.

There is some empirical evidence that alexithymia might not only be a consequence of victimization, but also a mediator to further negative outcomes. For example, in one study with 325 secondary school students, alexithymia partially mediated the relationship between peer victimization and deliberate self-harm (Garisch and Wilson, 2010). Along the same line, alexithymia mediated the association between traditional bullying victimization and post-traumatic stress symptoms in a sample of 488 high school students (Guzzo et al., 2014). More recently, Prino et al. (2019) have found in a sample of 1,092 fourth to sixth graders that alexithymia has mediated the relationship between verbal and relational bullying victimization and internalized/externalized symptoms.

### Understanding the Relationship Among Cyberbullying Victimization, Internet Addiction, and Alexithymia

Internet addiction is usually characterized by the following: (1) feeling a loss of time or a disregard of fundamental needs; (2) withdrawal, including negative emotions when the internet is not accessible; (3) an expanding need for more hours of internet use; and (4) negative psychological and social consequences due to internet use (Block, 2008). Associations between cyberbullying victimization and internet addiction can be explained as follows: For some cyberbullying victims, internet addiction can be considered a coping strategy to escape from unpleasant or the overwhelming feelings caused by cyberbullying. Thus, cyberbullying victims might lose the ability to recognize how much ICT use is appropriate. Cyberbullying victims might also overuse ICT because they spend excessive amounts of time using the internet to search for new comprising material spread by the cyberbullies. Along the same line, compulsive internet users had been shown to be socially isolated and lack social support and bonding, which is why they try to compensate for these social deficits by engaging in excessive online activities (Stodt et al., 2016). These characteristics are consistent with characteristics of cyberbullying victims who are excluded from peer activities and have fewer friends and positive peer relationships (Campbell et al., 2012; Wachs, 2012; Wright and Wachs, 2019). There is some empirical evidence based on cross-sectional and longitudinal studies that cyberbullying victims show higher risk for internet addiction (Mishna et al., 2012; Gámez-Guadix et al., 2013; Jung et al., 2014).

Alexithymic individuals have difficulties with developing healthy social relationships (Sifneos, 1973; Eichhorn et al., 2014; Karukivi and Saarijärvi, 2014). However, the online environment might be a suitable setting for alexithymic individuals to compensate for social deficits, because of the absence of physical presence and direct interaction with others, allowing these individuals to communicate with minimal direct interpersonal contact or a need to openly share emotions (Samur et al., 2013). Furthermore, the inability to modulate emotions through cognitive processes and impairments in emotional awareness might explain why alexithymic individuals tend to engage in impulsive actions to cope with unpleasant emotional states (Keltikangas-Järvinen, 1982). These actions are committed to discharge internal tension and can manifest themselves through compulsive behavior, such as eating disorders, gambling, or drug abuse (Keltikangas-Järvinen, 1982; Luminet et al., 1999; Morie et al., 2017). Therefore, it is reasonable to suggest that alexithymia is also related to internet addiction which can also be considered as a form of compulsive behavior performed to discharge internal tensions. There is some empirical research confirming a link between alexithymia and internet addiction (De Berardis et al., 2009; Craparo, 2011; Dalbudak et al., 2013; Kandri et al., 2014).

There is also some empirical evidence to support the assumption that alexithymia might also explain the relationship between cyberbullying victimization and internet addiction. For example, in one investigation based on 1,470 college students, alexithymia partially mediated the relationship between child maltreatment and internet addiction (Yates et al., 2012). Similarly, in another study with 358 high school students between the ages of 18 and 19, alexithymia mediated the relationship between a wide range of traumatic experiences (e.g., loss of a relative, serious physical harm) and internet addiction (Schimmenti et al., 2017).

## Aims of the Study

Based on the reviewed literature, there are reasons to expect that cyberbullying victimization is associated with lower self-esteem (Brighi et al., 2012; Brewer and Kerslake, 2015; Wachs et al., 2016; Palermiti et al., 2017; Lei et al., 2019) as well as higher alexithymia (Aricak and Ozbay, 2016). In addition, there is some evidence to suggest that higher alexithymia is correlated with lower self-esteem (Yelsma, 1995; Sayar et al., 2005; De Berardis et al., 2009; Sasai et al., 2010). There is also some evidence for an indirect effect between victimization and negative psychological health via alexithymia (Garisch and Wilson, 2010; Guzzo et al., 2014; Prino et al., 2019). However, until now, no study has investigated whether cyberbullying victimization is indirectly associated with self-esteem via alexithymia. Investigating possible indirect effects that explain the relationship among cyberbullying victimization and self-esteem via alexithymia might help to understand negative effects of cyberbullying victimization and underlying mechanism. Thus, the first research aim focused on examining the indirect association among cyberbullying victimization and self-esteem via alexithymia. We hypothesized:

H1: Cyberbullying victimization would be directly related to lower self-esteem.
H2: Cyberbullying victimization would be directly associated with greater alexithymia.
H3: Cyberbullying victimization would be indirectly associated with lower self-esteem via greater alexithymia.

As stated before, previous research has shown that cyberbullying victimization is associated with internet addiction (Gámez-Guadix et al., 2013; Jung et al., 2014). In addition, past research indicates that higher alexithymia is correlated with higher internet addiction (De Berardis et al., 2009; Craparo, 2011; Dalbudak et al., 2013; Kandri et al., 2014). Other work has shown that alexithymia mediates the association between traumatic experiences and internet addiction (Yates et al., 2012; Schimmenti et al., 2017). However, no study has investigated whether cyberbullying victimization is indirectly related to internet addiction via alexithymia. Investigating the indirect effect of cyberbullying victimization on Internet addiction and thus, explaining the mechanism that underlies this relationship might help to understand the damaging effects of cyberbullying victimization on adolescents and clarify which factors might further complicate recovery. Thus, the second research aim was to analyze the indirect relationship between cyberbullying victimization and internet addiction via alexithymia. We hypothesized:

H4: Cybervictimization would be directly associated with higher internet addiction.
H5: Cyberbullying victimization would be indirectly related to higher internet addiction via greater alexithymia.

**FIGURE 1:**
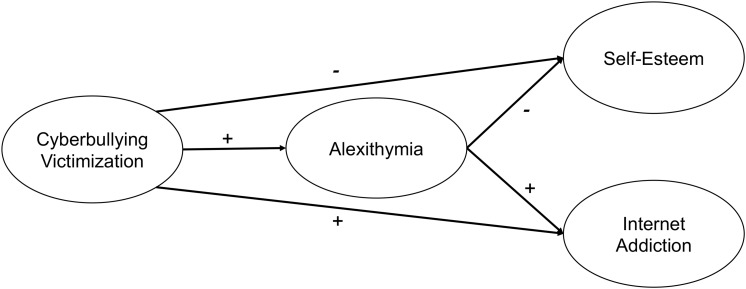
Conceptual model of direct and indirect associations among cyberbullying victimization, alexithymia, internet addiction, and self-esteem.

The conceptual model configuring the direct and indirect associations of the investigated variables is depicted in Figure 1.

## Materials and Methods

### Participants and Procedures

Self-reports of 1,442 adolescents from three schools in Germany, three schools in Netherlands, and one school in the United States were collected. Ages ranged between 12 and 17 years old [*M*_*age*_ = 14.17, *SD* = 1.38, 51.5% (*n* = 742) boys]. Regarding country of origin, the study sample included 847 German [50.2% (*n* = 425) boys], 371 Dutch [56.3% (*n* = 209) boys], and 224 American participants [48.2% (*n* = 108) boys]. Mean level comparisons of age of participants showed that the Dutch participants (*M*_*age*_ = 14.47, *SD* = 1.42) were significantly older than the German participants (*M*_*age*_ = 14.12, *SD* = 1.21) and the American participants (*M*_*a*__*ge*_ = 13.97, *SD* = 1.73). No sex differences by country of origin were found.

Ethical approval was granted by the educational authority of the federal state of Lower Saxony in Germany, as well as University Institutional Review Board in the United States. Schools were invited via email to take part in this study. In Germany and Netherlands, the data were collected by using an online questionnaire. In the United States, a paper-pencil questionnaire was implemented. The survey was completed during one regular school hour in the school’s computer lab and in classrooms, facilitated by trained research assistants. Parents of minors were required to sign a written consent form allowing adolescents to participate. Participants were told that they could stop the survey at any point if they wanted to. Answering the full questionnaire took around 30 min. Students were informed that they could seek out a school counselor or a trusted teacher for support to address emotional concerns at their schools.

### Measures

#### Cyberbullying Victimization

The questionnaire started with a definition of cyberbullying, which included the three central characteristics of cyberbullying (imbalance of power, repetition, and intention to hurt) and the use of ICT. In order to assess cyberbullying victimization, a scale consisting of four items, one item each for one form of cyberbullying, developed by Jäger et al. (2007), was used. For the assessment of cyber harassment, participants were asked “*How many times has someone sent you threats, defamations, or other aggravating messages via the internet/cell phone in the last twelve months?*”; for cyber denigration, “… *did someone spread rumors or defamations about you via the internet/cell phone*…*?*”; for cyber outing, “… *did someone hand on private emails, chat messages or pictures of you to others with the intention of exposing you*…*?*”; and for cyber exclusion, “…*someone excluded you from the group in chats or online games*…*?*” Participants answered using a five-point ordinal scale, “*Never*,” “*Once or twice*,” “*Twice or three times a month*,” “*About once a week*,” or “*Several times a week*.” Reliabilities were acceptable for the entire sample (α = 0.87), and the American (α = 0.71), the Dutch (α = 0.70), and German subsamples (α = 0.73).

#### Self-Esteem

Global self-esteem was measured by using the Rosenberg Self-Esteem Scale. This instrument consists of 10 items of general self-esteem including both positive and negative feelings about the self (Rosenberg, 1965). Participants rated how well statements, such as “*On the whole, I am satisfied with myself*” and “*I feel I do not have much to be proud of*,” described them on a four-point Likert scale from (“*strongly disagree*” to “*strongly agree*”). The higher participants scored on this scale the higher their self-esteem. Reliabilities were good for the entire sample (α = 0.85), and the American (α = 0.89), Dutch (α = 0.82), and German subsamples (α = 0.89).

#### Internet Addiction

To measure internet addiction, the internet-Related Experiences Questionnaire developed by Beranuy et al. (2009) was used. This scale consists of 10 items which include intra- and interpersonal conflicts due to ICT use. Participants rated how well statements, such as “*When you are not connected to the internet do you feel nervous or worried?*” and “*Do you get angry or irritated when someone distracts you while you are connected?*,” described their ICT use. Participants responded using a four-point ordinal scale: “never,” “rarely,” “sometimes,” or “often.” The higher participants scored on this scale the higher their internet addiction. Reliabilities were good for the entire sample (α = 0.82), and the American (α = 0.84), Dutch (α = 0.81), and German subsamples (α = 0.84).

#### Alexithymia

Emotional components of alexithymia were measured with the Toronto Alexithymia Scale (TAS-20) which originally includes three subscales: (1) difficulties identifying feelings (DIF), (2) difficulties in describing one’s feelings (DDF), and (3) externally-oriented thinking (EOT). Since the third subscale did not show good reliability among adolescents (Rieffe et al., 2006), we only assessed subscale 1 and 2 (12 items), which have shown good reliability and validity among adolescents (Heaven et al., 2010). In addition, other research has also shown that alexithymia can be reliable measured in adolescence using the TAS-20 without the EOT subscale (Loas et al., 2017; Preece et al., 2017). There is some empirical evidence that the two-factor model (DIF + DDF and EOT) provided acceptable model fit and had significant advantages over the three-factor model (DIF, DDF, and EOT) (Erni et al., 1997; Kooiman et al., 2002; Loas et al., 2017; Preece et al., 2017). Hence, we developed a combined sum score using the DIF and DDF subscales. Participants were asked to rate how much they agreed with the statements on a five-point Likert scale ranging from “strongly disagree” to “strongly agree.” The higher participants scored on this scale the higher their alexithymia. Cronbach’s alpha was excellent for the total sample (α = 0.90), and the American (α = 0.90), Dutch (α = 0.89), and German subsamples (α = 0.89).

#### Control Variables

Past research showed that age, sex, and cyberbullying perpetration is correlated with cyberbullying victimization and alexithymia (Tokunaga, 2010; Brody and Vangelisti, 2017; Wachs et al., 2017; Wachs and Wright, 2018).

Hence, we included adolescents’ age (years), sex (male; female), and cyberbullying perpetration as control variables. Cyberbullying perpetration was measured in the same way as cyberbullying victimization. Cronbach’s alpha was good for the total sample (α = 0.88), and the American (α = 0.87), Dutch (α = 0.83), and German subsamples (α = 0.79).

### Translation Procedure

The scale for measuring cyberbullying victimization was translated from German into Dutch and English, while the scale for measuring internet addiction was translated from English into German and Dutch. Appropriate back-translation techniques were applied. The self-esteem measure and subscales for measuring emotional components of alexithymia were available in all three languages.

### Data Analyses

Descriptive statistics and correlations were computed on all main study variables (self-esteem, internet addiction, and cyberbullying victimization). To investigate the direct and indirect associations between cyberbullying victimization, self-esteem, internet addiction, and alexithymia, mediation tests were completed in a structural equation modeling (SEM) framework using AMOS. It was hypothesized that cyberbullying victimization would predict self-esteem and internet addiction, mediated by alexithymia. Cyberbullying perpetration, participant age, and sex were entered as control variables in the model, predicting cyberbullying victimization and alexithymia. Mediation tests were conducted by using a bias-corrected bootstrapping procedure with 5,000 resamples. Latent constructs for cyberbullying victimization, internet addiction, self-esteem, and alexithymia were specified using two item parcels each, based on careful considerations of factor loadings in each scale. Specifically, items were assigned to parcels 1 and 2, respectively, where the highest loading item was assigned to the first parcel, the second highest item to the second one and so forth (Little et al., 2002).

Goodness-of-fit was examined by considering the following fit indices: The comparative fit index (CFI), Tucker–Lewis index (TLI), the root-mean-square error of approximation (RMSEA), and the standardized root-mean-square residual (SRMR). Model fit was evaluated using typical cut-off scores reflecting good fit to the data: CFI/TLI > 0.95 and.90; RMSEA < 0.06 and 0.08, and SRMR < 0.10 and 0.05 (Hu and Bentler, 1999).

Measurement invariance across the three countries was tested by comparing a freely estimated model to a constrained one, where paths from items to each respective latent construct were set to equality across groups. Results provided evidence that the scales measuring cyberbullying victimization (Δχ^2^ = 37.58, Δ*df* = 4, *p* < 0.001), alexithymia (Δχ^2^ = 48.56, Δ*df* = 12, *p* < 0.001), self-esteem (Δχ^2^ = 87.78, Δ*df* = 10, *p* < 0.001), and internet addiction (Δχ^2^ = 21.99, Δ*df* = 10, *p* < 0.015) varied across the groups. Therefore, subsequent model tests were tested separately in the American, Dutch, and German samples.

## Results

### Descriptive Analyses

Descriptive statistics and bivariate correlations of all study variables are included in Table 1. All correlations were in the expected direction. Higher levels of cyberbullying victimization were positively associated with alexithymia and internet addiction as well as negatively associated with self-esteem.

**TABLE 1:** Descriptive statistics and correlations.

Measure	1	2	3	4	*M*	*SD*
Cyberbullying victimization	–				1.34	0.650
Alexithymia	0.296**	–			2.18	0.961
Self-esteem	−0.233**	−0.491**	–		17.91	5.78
Internet addiction	0.220**	0.317**	−0.228**	–	2.12	0.649

**p < 0.01.

### Structural Equation Modeling

Model test provided the following evidence: In the American sample, the model also had acceptable fit to the data: χ^2^ = 89.16 *df* = 46, *p* < 0.001, CFI = 0.951, TLI = 0.917, RMSEA = 0.065 [90% CI = 0.044, 0.085], *p* close = 0.109, SRMR = 0.058. Findings provided evidence of that the direct effect of cyberbullying victimization on self-esteem did not reach statistical significance (β = −0.11; *p* = 0.133). Additionally, a positive direct effect of cyberbullying victimization on alexithymia (β = 0.29; *p* < 0.001) and on internet addiction (β = 0.27; *p* = 0.001) was found. Moreover, a negative direct effect of alexithymia on self-esteem (β = -0.60; *p* < 0.001) and a positive direct effect of alexithymia on internet addiction (β = 0.28; *p* < 0.001) was found. The indirect effects from cyberbullying victimization to self-esteem (β = -0.20; 95% CI = −0.31, −0.11) and internet addition (β = 0.08; 95% CI = 0.04, 0.16), mediated by alexithymia, were both statistically significant. The tested model explained 10.4% of variance in alexithymia (*R*^2^ = 0.10), 21.0% in internet addiction (*R*^2^ = 0.21), and 41.1% in self-esteem (*R*^2^ = 0.41).

In the Dutch sample, the model had a good fit: χ^2^ = 88.35 *df* = 46, *p* < 0.001, CFI = 0.970, TLI = 0.949, RMSEA = 0.050 [90% CI = 0.034, 0.065], *p* close = 0.483, SRMR = 0.048. Findings indicated a negative direct effect of cyberbullying victimization on self-esteem (β = -0.14; *p* = 0.032), a positive direct effect of cyberbullying victimization on alexithymia (β = 0.41; *p* < 0.001), and on internet addiction (β = 0.18; *p* = 0.011). Additionally, a negative direct effect was found of alexithymia on self-esteem (β = -0.49; *p* < 0.001) as well as a positive direct effect of alexithymia on internet addiction (β = 0.35; *p* < 0.001). The indirect effects of cyberbullying victimization on self-esteem (β = -0.20; 95% CI = -0.28, -0.14) and internet addition (β = 0.15; 95% CI = 0.09, 0.23), mediated by alexithymia, were both statistically significant. The model explained 24.2% of variance in alexithymia (*R*^2^ = 0.24), 21.1% in internet addiction (*R*^2^ = 0.21), and 32.6% in self-esteem (*R*^2^ = 0.33).

In the German sample, the model had good fit: χ^2^ = 142.91 *df* = 46, *p* < 0.001, CFI = 0.975, TLI = 0.958, RMSEA = 0.050 [90% CI = 0.041, 0.059], *p* close = 0.490, SRMR = 0.037. Results showed that the direct effect of cyberbullying victimization on self-esteem did not reach statistical significance (β = −0.04; *p* = 0.215). Furthermore, a positive direct effect of cyberbullying victimization on alexithymia (β = 0.32; *p* < 0.001) and on internet addiction (β = 0.22; *p* < 0.001) was found. Additionally, a negative direct effect of alexithymia on self-esteem (β = -0.63; *p* < 0.001) and a positive direct effect of alexithymia on internet addiction (β = 0.36; *p* < 0.001) was found. The indirect effects from cyberbullying victimization on self-esteem (β = −0.20; 95% CI = −0.26, −0.15) and internet addition (β = 0.12; 95% CI = 0.08, 0.16), mediated by alexithymia, were both statistically significant. The tested model explained 20.6% of variance in alexithymia (*R*^2^ = 0.21), 23.2% in internet addiction (*R*^2^ = 0.23), and 41.6% in self-esteem (*R*^2^ = 0.42).

## Discussion

The purpose of the present study was to add to the empirical evidence on the effects of cyberbullying victimization on self-esteem and internet addiction via alexithymia from a trauma theoretical perspective. The findings contribute to our understanding of how cyberbullied adolescents can be effectively supported, clarify which factors might further complicate recovery from cyberbullying victimization, and, thus, help to develop tertiary cyberbullying prevention strategies and identify the needs of cyberbullied adolescents.

### Cyberbullying Victimization, Self-Esteem, and Alexithymia

Contrary to study hypothesis 1 (H1) and previous research (Brighi et al., 2012; Brewer and Kerslake, 2015; Wachs et al., 2016; Palermiti et al., 2017), the analyses showed that only in the Dutch sample, but not in the American and German samples, a direct association between cyberbullying victimization and self-esteem was found. It is not entirely clear why these inconsistencies were found. More cross-national comparisons on this association are needed to better understand it.

Consistent with expectations, cyberbullying victimization was positively associated with alexithymia (H2). This finding was made across all three country samples which was consistent with some prior work on the relationship between traditional and cyberbullying victimization and alexithymia (Garisch and Wilson, 2010; Guzzo et al., 2014; Aricak and Ozbay, 2016; Prino et al., 2019). One potential explanation for this finding includes that alexithymia evoked through cyberbullying victimization is a coping mechanism to suppress and deny painful emotions resulting from cyberbullying victimization.

Consistent with hypothesis 3 (H3), evidence for an indirect effect of higher cyberbullying victimization on lower self-esteem via greater alexithymia was found. This finding extends related research which showed that alexithymia mediates the associations between victimization and post-traumatic stress symptoms (Guzzo et al., 2014), deliberate self-harm (Garisch and Wilson, 2010), and externalizing/internalizing problems (Prino et al., 2019).

Study findings also revealed that alexithymia was negatively associated with self-esteem which is consistent with research among young adults (Yelsma, 1995; De Berardis et al., 2009; Sasai et al., 2010) and work among Turkish adolescents (Sayar et al., 2005). One possible explanation is that alexithymic individuals show not only emotional awareness, but also experience a lack of social attachment, poor interpersonal relating, and social skills. Since interpersonal relating and social skills are also crucial for developing a positive self-view, these characteristics might also interfere with establishing positive self-esteem.

### Cyberbullying Victimization, Internet Addiction, and Alexithymia

As hypothesized, across all three country samples, cyberbullying victimization was positively associated with internet addiction (H4) which was consistent with other research (Mishna et al., 2012; Gámez-Guadix et al., 2013; Jung et al., 2014). We propose that cyberbullying victims lose their sense of an appropriate use of ICT and control over their ICT use, try to compensate for social deficits in the real world, or try to discharge internal tensions caused by cyberbullying victimization that manifest through compulsive internet behaviors.

Finally, consistent with study expectations, we found evidence for an indirect effect of cyberbullying victimization on higher internet addiction via greater alexithymia (H5) across all three samples. Broadly speaking, this finding is consistent with previous work which has found that alexithymia partially mediated the effect of child maltreatment on internet addiction (Yates et al., 2012) and the association between traumatic experiences and internet addiction (Schimmenti et al., 2017). Further, the evidence showed that alexithymia was positively associated with internet addiction, again consistent with previous empirical work on young adults (De Berardis et al., 2009; Dalbudak et al., 2013; Kandri et al., 2014). It is likely that alexithymic adolescents might try to compensate for social deficits by utilizing ICTs. There is some evidence that alexithymic individuals prefer the online environment to communicate and interact with others due to the absence of the direct interactions with others (Samur et al., 2013). In addition, the inability to modulate emotions through cognitive processes in alexithymic individuals might increase impulsive actions which are committed to discharge internal emotional states or tension and can manifest through a variety of compulsive behaviors (Keltikangas-Järvinen, 1982; Luminet et al., 1999; Morie et al., 2017). Internet addiction might be one way to discharge these internal tensions.

Taken together, analyzing indirect relationships between cyberbullying victimization and self-esteem and cyberbullying victimization and internet addiction across the three country samples provides evidence that alexithymia plays an important role in understanding possible consequences on adolescent psychological health and well-being and appears to contribute to maladaptive coping.

### Limitations

Even though the present study contributes information on the indirect effects of alexithymia on the relationship between cyberbullying victimization and self-esteem as well as internet addiction, a number of limitations require mention. Due to the cross-sectional nature of the present study, no causality can be inferred. Therefore, temporal ordering between cyberbullying victimization, self-esteem, internet addiction, and alexithymia cannot be determined. Longitudinal research with at least three measurement points is needed to further substantiate the mediating relationships tested in the present study. Furthermore, the data were exclusively collected through self-reports. Therefore, the observed relationships might be inflated due to shared method variance. A multi-informant approach could overcome this limitation in future research. Although there is some empirical evidence that alexithymia among adolescents can be reliably measured with the TAS-20 without using the EOT subscale (Loas et al., 2017; Preece et al., 2017), more research is needed that also considers the indirect effects of EOT on the relationship among cyberbullying victimization, self-esteem, and internet addiction. Lastly, although our sample is large enough to investigate cyberbullying victimization and its correlates, it cannot be considered as representative, and a relatively small number of schools were recruited. Therefore, findings should be interpreted with this in mind.

### Practical Implications

The finding that cyberbullying victimization is indirectly associated with lower self-esteem indicates that it is important to empower adolescent at-risk for cyberbullying victimization through prevention efforts. As previously described, self-esteem is a social construct which depends in particular during adolescence on interactions with peers. Therefore, it seems to be important to provide cyberbullying victims with opportunities to socialize with peers, build good peer relations as well as high quality friendships that help them value themselves and build a positive self-image.

Since cyberbullying victimization is positively associated with internet addiction adolescents need to be educated with the age-appropriate information they need to make sensible, informed choices about their internet use. The internet addiction scale used in the present study reflects aspects of intrapersonal conflicts associated with internet addiction (i.e., get angry when someone distracts online time), but also interpersonal conflict associated with internet addiction (i.e., easier to make new friends online and the impression that it is more comfortable to relate to people online than offline). Therefore, it appears to be imperative to offer adolescents meaningful leisure outlets where they can enjoy “offline time” and associate with peers.

Study findings also provide evidence that alexithymia might play a crucial role in understanding the relationship between cyberbullying victimization, self-esteem, and internet addiction. Therefore, it appears to be important to include emotional intelligence training (i.e., teaching the necessity and usefulness of emotions in human’s life, educating how to understand one’s emotions, and teaching words and ways to express positive and negative emotions) in cyberbullying prevention and intervention programs, but also in regular school curricula.

## Conclusion

In conclusion, the present study further advances our understanding of the negative effects of cyberbullying victimization on psychosocial and behavioral adjustment among adolescents. More specifically, this investigation highlights the role of alexithymia in understanding the associations among cyberbullying victimization, self-esteem, and internet addiction. The evidence shows that alexithymia functions as a psychological mechanism by which adolescents manage to cope with overwhelming affect resulting from cyberbullying victimization. Thus, findings support the importance of including emotional intelligence training in cyberbullying prevention and intervention efforts. The study findings show that cyberbullying victimization is related to lower self-esteem, higher internet addiction, and alexithymia; thus, it appears to be important that cyberbullying victims are monitored for these difficulties.

## Data Availability Statement

The raw data supporting the conclusions of this article will be made available by the authors, without undue reservation, to any qualified researcher.

## Ethics Statement

The studies involving human participants were reviewed and approved by the University Institutional Review Board (13-0962-P4J), University of Kentucky. Written informed consent to participate in this study was provided by the participants’ legal guardian.

## Author Contributions

SW designed this study and drafted the manuscript. GK, AV, and SW performed the statistical analyses. MW, GK, and AV provided constructive and editorial feedback on drafts of the manuscript. All authors read and approved of the manuscript.

## Conflict of Interest

The authors declare that the research was conducted in the absence of any commercial or financial relationships that could be construed as a potential conflict of interest.
